# Vaginal atresia and cervical agenesis combined with asymmetric septate uterus: A case report of a new genital malformation and literature review

**DOI:** 10.1097/MD.0000000000009674

**Published:** 2018-01-19

**Authors:** Tian-Tian Han, Juan Chen, Shu Wang, Lan Zhu

**Affiliations:** Department of Obstetrics and Gynecology, Peking Union Medical College Hospital, Chinese Academy of Medical Sciences and Peking Union Medical College, Beijing, China.

**Keywords:** cervical agenesis, congenital female genital malformation, septate uterus, vaginal atresia

## Abstract

**Rationale:**

A combination of Vaginal atresia and septate uterus as a novel genital malformation has been reported the first time. It offers a support for the bidirectional theory.

**Patient concerns:**

A 23-year-old woman presented with the inability to perform intercourse. The unprecedented variant was misdiagnosed by magnetic resonance imaging and ultrasonography as low vaginal atresia associated with complete septate uterus with functional endometrium in both the cavities.

**Diagnoses:**

The correct diagnosis was made and confirmed intraoperatively as a genital malformation, vaginal atresia and cervical agenesis associated with septate uterus.

**Intervention:**

laparoscopic and hysteroscopic diagnosis the novel malformation never been reported and a vaginal stent was recommended postoperatively.

**Outcomes:**

This rare clinical variant made misdiagnosis. Intraoperatively, unprecedented genital malformation was confirmed. There are no vaginal atresia cases in the literature with a septate functional uterus and single agenesis cervix.

**Lessons:**

Confirmed diagnosed by operation instead of depending on the imaging should be used for rare genital anomaly.

## 1 Introduction

Vaginal atresia, a rare Müllerian anomaly, with an incidence of 1/4000 to 1/10000 persons, is frequently characterized by a normal lower vagina or the absence of a vagina.^[1]^ Vaginal atresia can present as distal or total vaginal atresia, and in the latter case, the uterus always has an anatomically normal corpus and cervical aplasia, except in sporadic patients, in whom a rudimentary horn accompanies the uterus.^[2,3]^ The case described herein is a new female genital anomaly with a combination of vaginal atresia and cervical agenesis with septate uterus.

## 2 Case report

**Figure 1:**
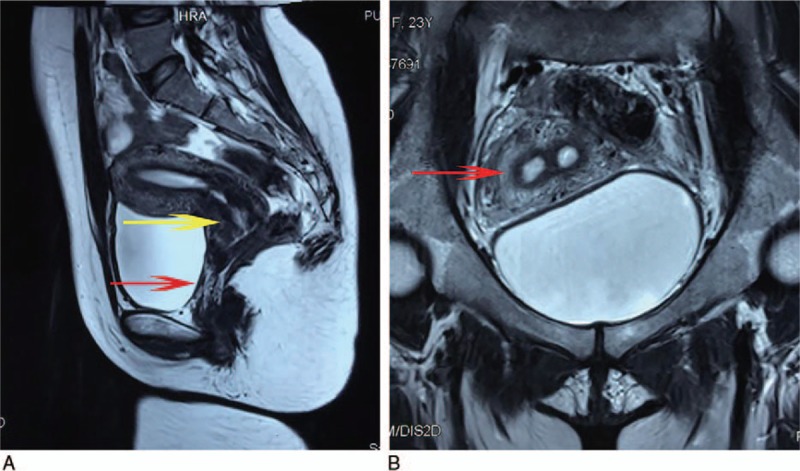
Magnetic resonance imaging scan showing (A) distinct cervices (yellow arrow) and no obvious low vagina (red arrow). (B) The septum is causing exaggerated separation of the cavities (red arrow).

**Figure 2:**
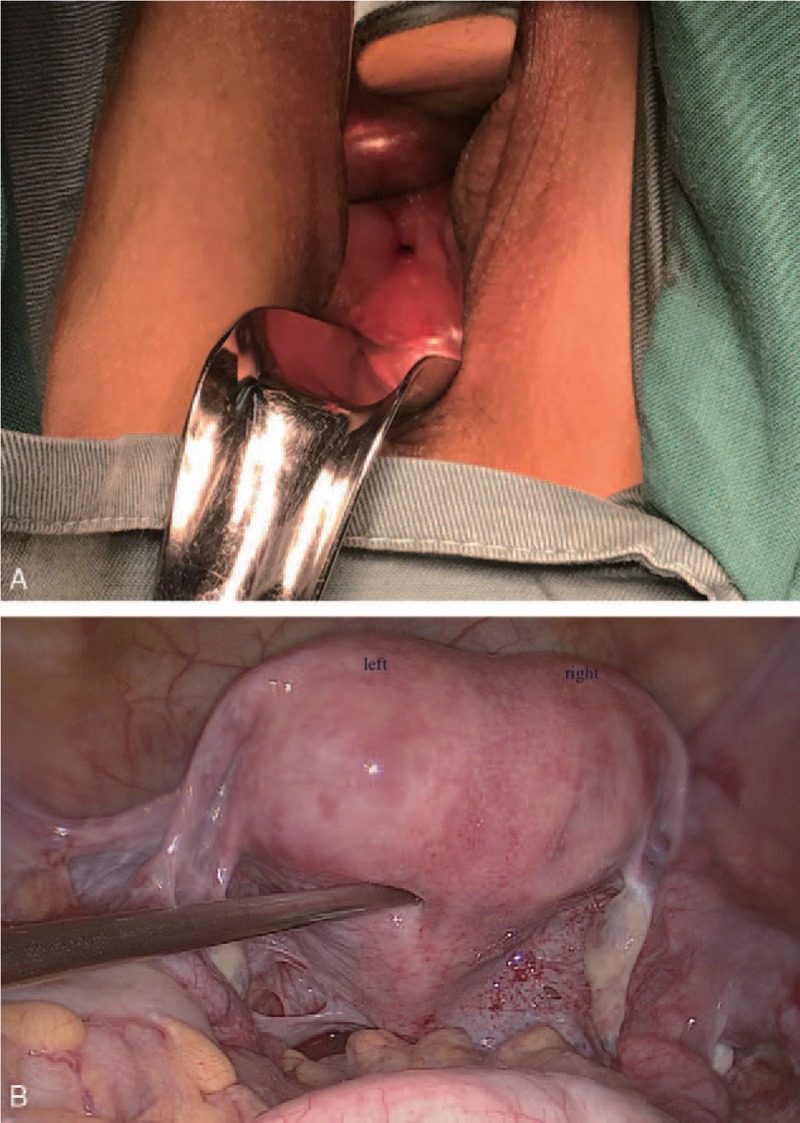
Surgical view (A) short vagina with a pored cervical structure at the top. (B) Laparoscopic view of the enlarged uterus with an asymmetric, small indentation at the top.

A 23-year-old woman whose complaint was dyspareunia underwent vaginoplasty 10 years ago at a local hospital for acute abdominal pain without menarche, and she had been suspected to have vaginal atresia, although the uterine malformation had not been detected during initial clinical management. Postoperative menstrual function was normal without dysmenorrhea. In August 2017, she was referred to our hospital for difficulty performing intercourse. She had no other complaints, such as abdominal pain during her menstrual cycle, an irregular menstrual cycle, or obstructed menstrual fluid flow. The pelvic ultrasonograms illustrated a complete septum arising from the fundus and extending to the endocervical os with a flat external uterine contour. The echogenic images within the septum showed that the septum measured 15 mm at its greatest diameter. For further evaluation, she was examined preoperatively with magnetic resonance imaging; the scan showed distended endometrial cavities and cervical canals, which were misdiagnosed as a lower obstructed vagina, 1-cm long duplication of a normal cervical zonal anatomy, and complete septate uterus (Fig. 1). There were no abnormal findings in the urinary system and hormonal profile. Breast and pubic hair were categorized as Tanner stage 5. The clinical examination of the spine and limbs was normal. On physical examination, she was found to have no development of hymenal remnant tissue and a vagina of adequate caliber but only 3 cm long at the apex, of which there was patency with one fingerbreadth at the site of the suspected vaginal stricture secondary to the first operation (Fig. 2A).

To resolve the stricture, the patient was placed under anesthesia. The vaginal segment was firm and 1-cm thick, full of fibrous, muscular connective tissue, but absent of endocervical and ectocervical components, as confirmed pathologically. An index finger was probed into the hole, and the cervical structure could not be reached; however, a longitudinal septum segmented a cavity into 2 parts. Then we switched to hysteroscopy. There was a 3-cm long longitudinal septum separating the normal endometrium cavity into an asymmetric hemiuterus: the right side was narrower than the left part morphologically, and the tubal ostias could be seen. Laparoscopically, the uterus was single with a round ligament attached bilaterally and enlarged, and the fundal contour was a small, smooth indentation. The speculum examination showed the ovaries and fallopian tubes, which were unremarkable, but the structure of the cervical ligament was unclear (Fig. 2B).

**Figure 3:**
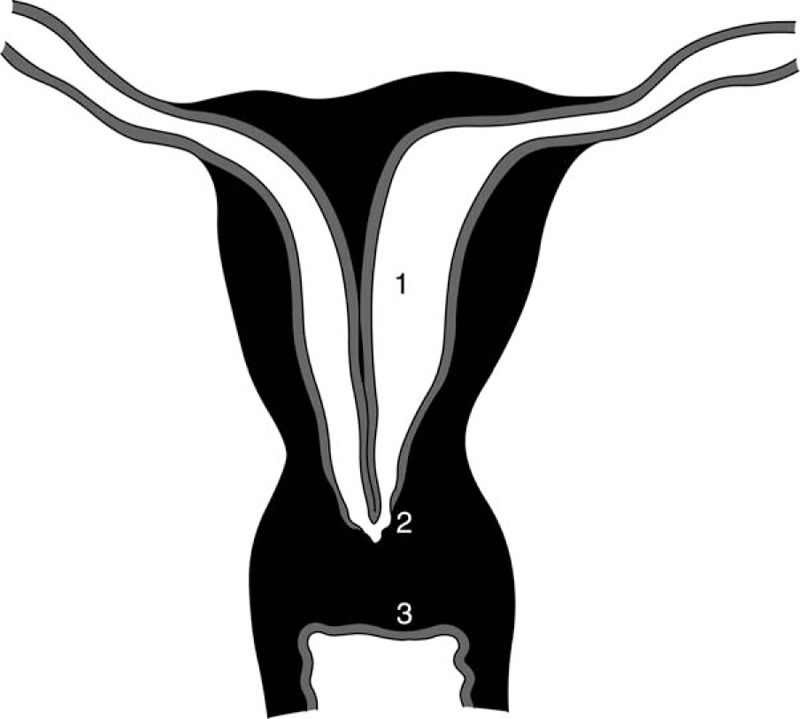
Features of the patient's condition: (1) complete septate uterus with asymmetric cavities, (2) agenesis cervix with a small hole, and (3) upper vaginal atresia (punched and shaped 10 years ago).

In this patient, the congenital combination of upper vaginal atresia, cervical agenesis, and complete uterine septum was identified (Fig. 3). For the atretic vagina, a vaginal stent was recommended to apply on daily self-dilatation postoperatively, and she experienced satisfactory intercourse in a short-term follow-up visit.

Written informed consent was obtained from the patient.

## 3 Discussion

We described a case of a genital anomaly that has not been previously reported in the literature. The classification systems of the American Fertility Society do not include this condition^[4]^; however, it can be classified by the European Society of Human Reproduction and Embryology and the European Society for Gynaecological Endoscopy system released in 2013 as U2bC4V4.^[5]^

The embryology of this complicated spectrum of genital malformations is controversial because of the questionable developmental progress. Based on the classic unidirectional theory,^[6]^ the fusion and canalization of Müllerian ducts progresses in a caudad-to-cranial direction, which makes it impossible to explain how the complete septate uterus developed in the absence of the cervix. However, the bidirectional theory^[6,7]^ can explain the combination of a septate uterus, double cervix, and longitudinal vaginal septum. According to this theory, the fusion and canalization of Müllerian ducts start from the isthmus, and then proceeds in both directions separately. Our case might provide additional evidence to support that the fusion of the upper part of the Müllerian ducts can be complete, but subsequent resorption of the median septum does not occur, resulting in formation of a complete septate corpus. Simultaneously, at the lower part of the Müllerian duct, the fusion progress does not occur or anomalous development results in cervical agenesis and upper vaginal atresia.

The literature, without date limitation, was reviewed in terms of vaginal agenesis or vaginal atresia by searching PubMed for the association with functional uterine anomalies (Table 1). Congenital vaginal atresia occurs as an isolated Müllerian anomaly or as a part of a syndrome.^[5,8]^ Vaginal agenesis is not the same entity as distal vaginal atresia. Only 2% to 7% of patients with partial or total vaginal aplasia have an active endometrium in uterine structures.^[8,9]^ However, in the latter, normal uterine and fallopian tubes exist.^[10]^ Sporadic cases of vaginal atresia combined with a functional uterine anomaly have been reported,^[11,12]^ but no concurrence of septate uterus has been reported (Table 1), although the septate uterus composes approximately 53.7% to 55% of all female genital anomalies.^[13,14]^ In conclusion, such a case is significant for clinical management and emerges as a novel anomaly that supports one embryologic concept. It may be necessary to determine the malformation during sterile operation. Also it is of crucial importance for us to observe the patient well into reproductive and obstetric outcomes in future.

**Table 1:**
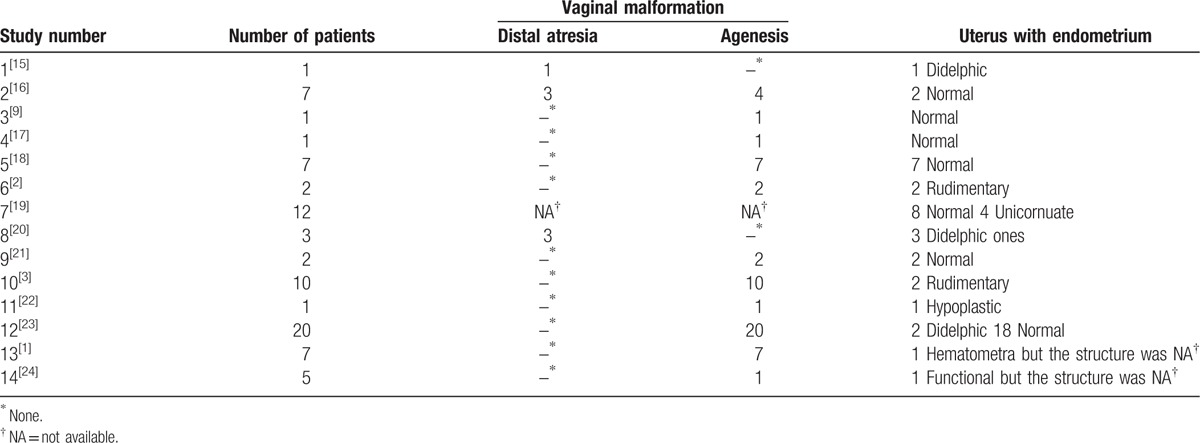
Review of literatures on vaginal agenesis or atresia with functional uterus.
